# Aging exacerbates mortality of *Acinetobacter baumannii* pneumonia and reduces the efficacies of antibiotics and vaccine

**DOI:** 10.18632/aging.101495

**Published:** 2018-07-18

**Authors:** Hao Gu, Dong Liu, Xi Zeng, Liu-Sheng Peng, Yue Yuan, Zhi-Fu Chen, Quan-Ming Zou, Yun Shi

**Affiliations:** 1 National Engineering Research Center of Immunological Products, Department of Microbiology and Biochemical Pharmacy, College of Pharmacy, Army Medical University, Chongqing 400038, PR China

**Keywords:** *Acinetobacter baumannii*, aging, pneumonia, inflammation

## Abstract

Pneumonia caused by *Acinetobacter baumannii* has become a serious threat to the elderly. However, there are no experimental studies on the relevance between aging and *A. baumannii* infections. Here, we established an aged pneumonia mouse model by non-invasive intratracheal inoculation with *A. baumannii*. Higher mortality was observed in aged mice along with increased bacterial burdens and more severe lung injury. Increased inflammatory cell infiltration and enhanced pro-inflammatory cytokines at 24 hours post infection were detected in aged mice than those in young mice. Moreover, infected aged mice had lower myeloperoxidase levels in lungs and less reactive oxygen species-positive neutrophils in bronchoalveolar lavage fluid compared with infected young mice. Reduced efficacy of imipenem/cilastatin against *A. baumannii* was detected in aged mice. Vaccination of formalin-fixed *A. baumannii* provided 100% protection in young mice, whereas the efficacy of vaccine was completely diminished in aged mice. In conclusion, aging increased susceptibility to *A. baumannii* infection and impaired efficacies of antibiotics and vaccine. The aged mice model of *A. baumannii* pneumonia is a suitable model to study the effects of aging on *A. baumannii* infection and assess the efficacies of antibiotics and vaccines against *A. baumannii* for the elderly.

## Introduction

*Acinetobacter baumannii* has emerged as an important pathogen of both community-associated and nosocomial infections worldwide. This bacterium can cause many types of infections, including pneumonia, bacteremia, meningitis, urinary tract infection, and wound infection. Among these diseases, the most common is pneumonia [1,2]. *A. baumannii* infections have become increasingly difficult to treat because most isolates are highly resistant to a wide range of antibiotics, displaying multidrug-resistant (MDR) or extensively drug-resistant. A retrospective cohort study including 175 hospitals in U.S. showed the prevalence of MDR-strains among patients with *A. baumannii* infection is > 80% [3]. In 2017, WHO published its first ever list of 12 families of antibiotic-resistant "priority pathogens" to help in prioritizing the research and development of new and effective antibiotic treatments. Carbapenem-resistant *A. baumannii* has been listed as top one of the most critical resistant bacteria which are in an urgent need for the new treatment [4]. Patients infected with MDR *A. baumannii* have significantly prolonged hospital stays than those infected with drug-sensitive strains. What’s more, infections caused by MDR *A. baumannii* are associated with a tendency to higher mortality rates [5–7].

Clinical data indicate that aging is a risk factor for *A. baumannii* infection [8]. A 6-year period study on *A. baumannii* infection showed that 68% of the patients are aged over 60 years [9]. Another research showed aging is significantly associated with in-hospital mortality in *A. baumannii* infection [10]. More importantly, the elderly have worse outcomes than the young after *A. baumannii* infection [11,12]. But there is no experimental data supporting these observations due to the lack of aged mouse model of *A. baumannii* infection.

The world population is rapidly aging and 1/3 deaths in the elderly are due to infection [13], so preventing *A. baumannii* infection in the elderly would be an important public health issue. Aged people have dysregulated adaptive and innate immune systems [14–16], which might influence the prevention and treatment of *A. baumannii* infection. Studies from influenza vaccine or herpes zoster vaccine showed protection induced by immunizations is reduced in the elderly compared with the adults [17,18]. It has been shown that *Clostridium difficile*-infected aged mice treated with vancomycin are more susceptible to relapse than young mice [19], indicating the different response to antibiotics in aged mice. Currently, most of the drug and vaccine evaluations were performed in young or adult mice and population, which might be not suitable to be transferred to the clinical use for the elderly. Therefore, developing antibiotics or vaccines against *A. baumannii* should consider the effect of aging and the efficacies should be evaluated in aged models in preclinical research to make sure they also work for the elderly. But there is a lack of an aged *A. baumannii*-infected animal model to perform such studies.

In this study, we built an aged mice model with respiratory infection of *A. baumannii* and compared the outcomes and host response between aged and young mice. We further tested the efficacies of antibiotics and vaccine in aged mice and tried to figure out whether aging affects their efficacies.

## RESULTS

### Enhanced mortality in aged mice after respiratory *A. baumannii* infection

**Figure 1:**
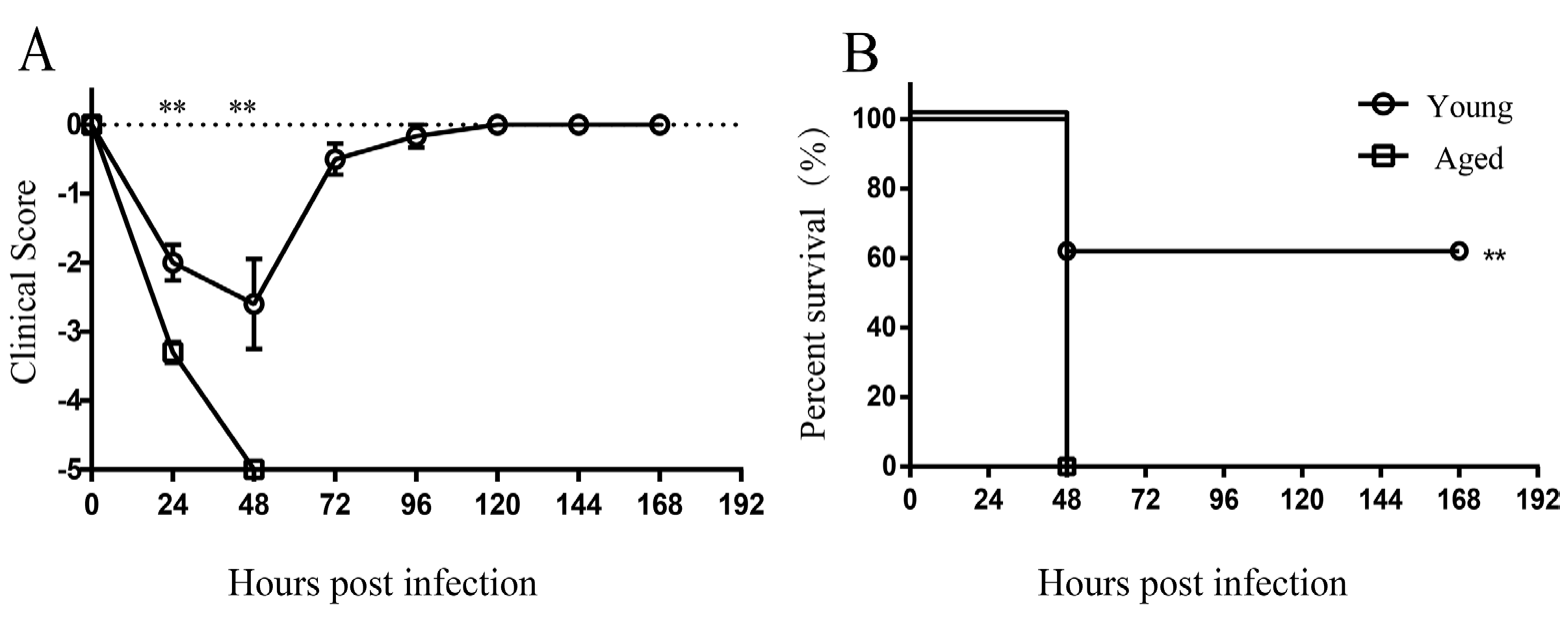
**Increased susceptibility to *A. baumannii* infection in aged mice.** Young (n = 10) and aged mice (n = 10) were infected intratracheally with 5×10^6^ CFU of LAC-4. (**A**) Clinical score and (**B**) survival rate of mice were monitored for 7 days. Clinical score is expressed as means ± SEM. The data represent one of 2 independent experiments. Survival curves were compared using log-rank test. Statistical significance of clinical score was determined by Student’s *t* test. **, *P* < 0.01.

To determine whether susceptibility to *A. baumannii* infection was increased with advanced age, young and aged mice were infected by non-invasive intratracheal inoculation of LAC-4 (5×10^6^ CFU). Survival rate and clinical score of mice were monitored for 7 days. After infection, the clinical signs of aged mice were more severe than those of young mice (Figure 1A). All aged mice appeared very sick, moved slowly, and hunched at 24 hours post infection (hpi). Mortality of aged mice was 100% within 48 hours after LAC-4 infection, whereas 60% of young mice survived (Figure 1B). The results indicate aged mice are more susceptible to respiratory *A. baumannii* infection than young mice.

### Increased bacterial burdens and lung injury in aged mice

**Figure 2:**
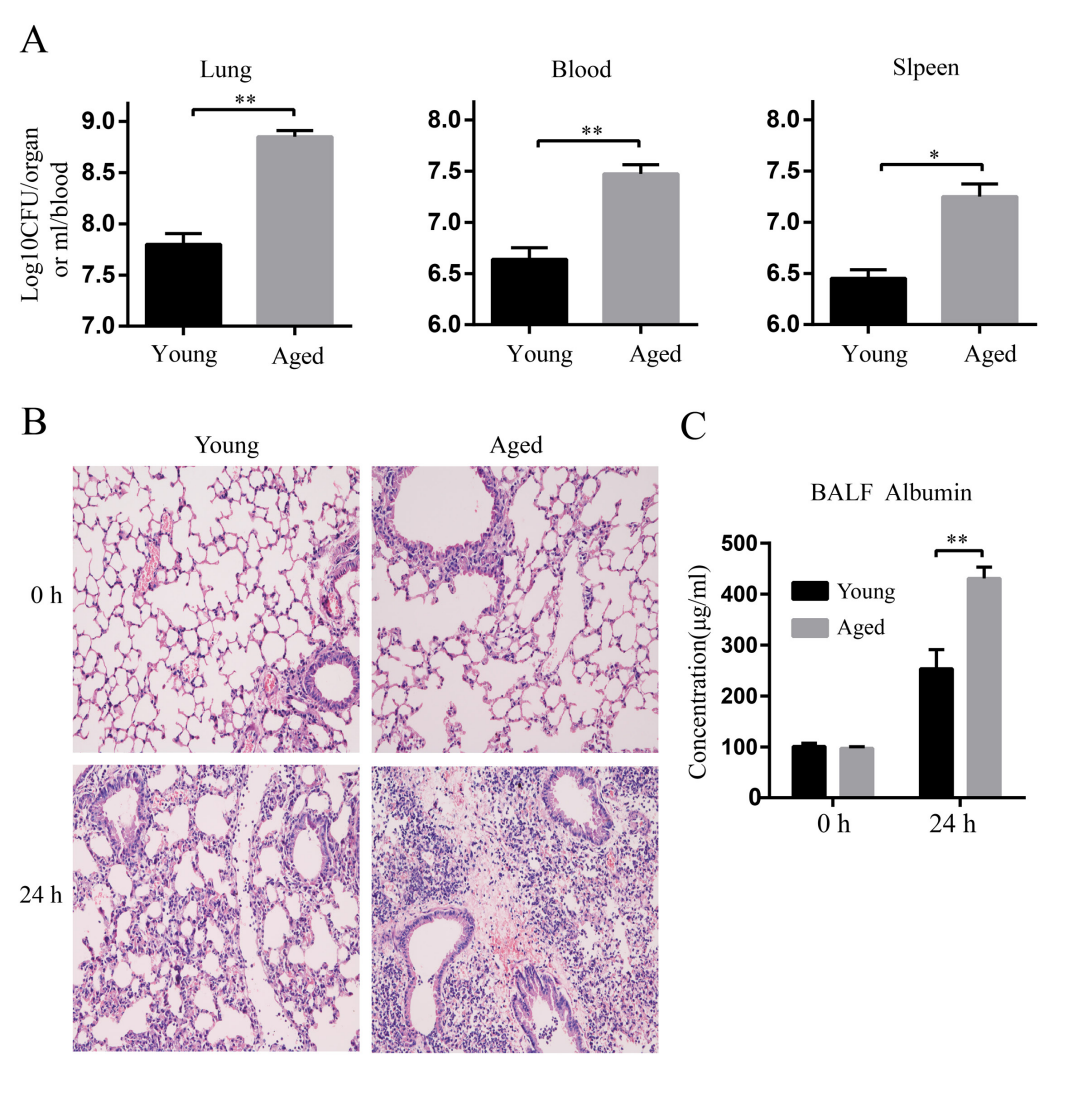
**Increased bacterial burdens and lung damage after *A. baumannii* infection in aged mice.** Young and aged mice were infected intratracheally with 5×10^6^ CFU of LAC-4 and the lung, blood, and spleen were collected at 0 h and 24 hpi. (**A**) Bacterial burdens in the lung, blood, and spleen of young and aged mice at 24 hpi were counted on TSA plates. (**B**) Lung histopathology of young and aged mice were observed at 0 h and 24 hpi (magnification=200×). (**C**) Serum albumin levels in BALF of young and aged mice were determined by ELISA. Data are presented as mean ± SEM of five mice per condition and represent one of 2 independent experiments. Statistical analyses were performed by Student’s *t* test. *, *P* < 0.05, **, *P* < 0.01.

Next, bacterial burdens in the lung, blood, and spleen at 24 hpi were evaluated. Aged mice had more than 10-fold higher bacterial burdens in lungs compared with young mice. More importantly, aged mice had remarkably increased bacterial burdens in blood and spleen compared with young mice, showing higher level of extrapulmonary dissemination of bacteria after infection (Figure 2A).

We next evaluated lung injury after LAC-4 infection by histological analysis. As shown in Figure. 2B, the lung histology of aged mice was comparable to that of young mice before infection (0 h). Whereas, histopathological changes of lungs in aged mice were more severe than those in young mice at 24 hpi. Lungs of young mice showed limited tissue destruction with mild infiltration of perivascular and interstitial inflammatory cells. In contrast, lungs of aged mice appeared more severe and extended lesions with extensive accumulation of inflammatory cells (Figure 2B). Serum albumin in bronchoalveolar lavage fluid (BALF) is an indicator for assessing the epithelial barrier permeability of lung. Aged mice exhibited higher levels of serum albumin in BALF at 24 hpi than young mice (Figure 2C), indicating that the epithelial barrier of aged mice was destroyed more severely than that of young mice after infection. These results suggest *A. baumannii* infection causes more severe lung damage in aged mice.

### Elevated inflammatory cytokine responses in aged mice after *A. baumannii* infection

**Figure 3:**
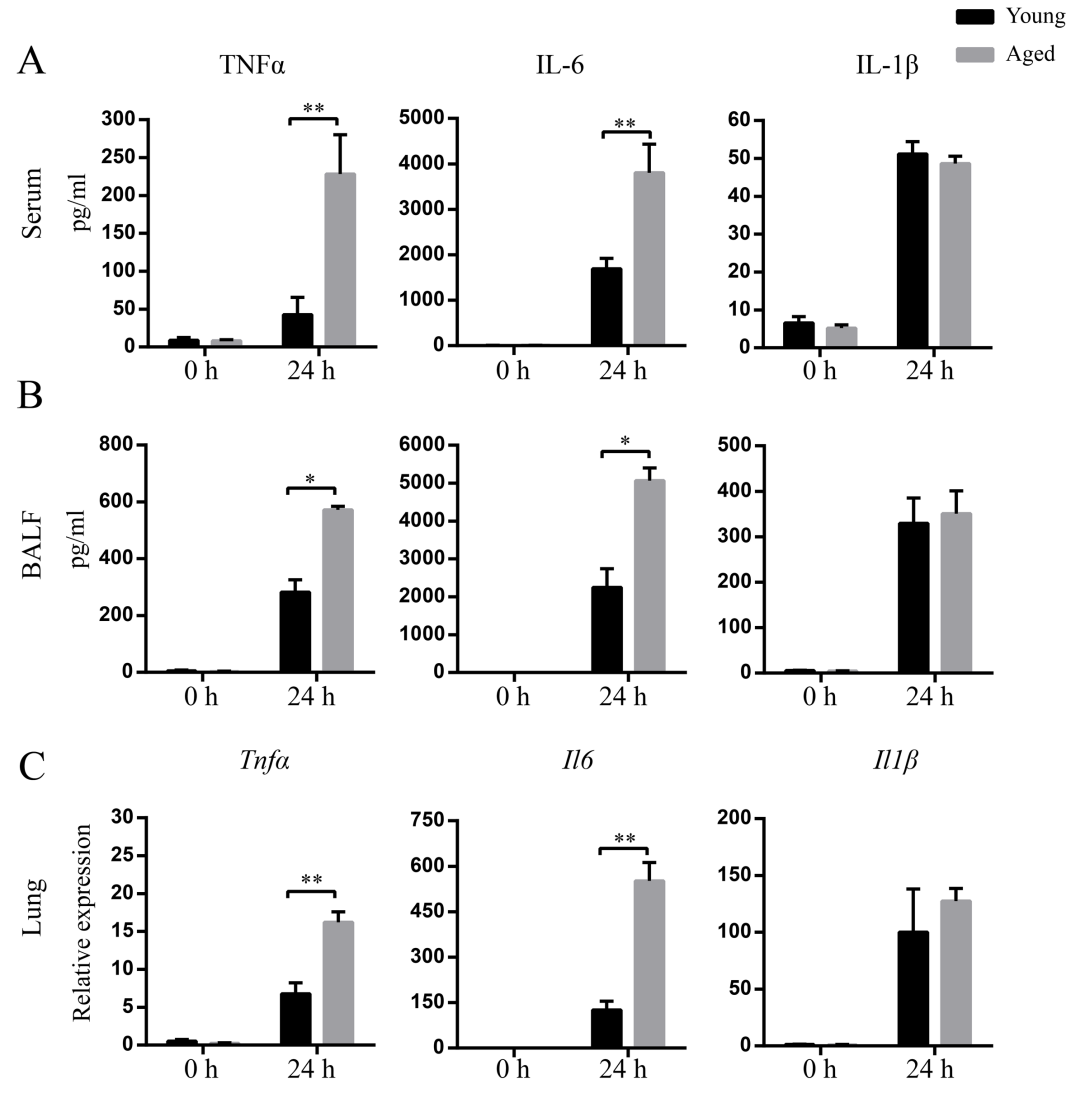
**Enhanced levels of cytokines after *A. baumannii* infection in aged mice.** Young and aged mice were infected intratracheally with 5×10^6^ CFU of LAC-4. At 0 h and 24 hpi, serum and BALF were collected and mRNA was isolated from lungs. TNFα, IL-6, and IL-1β levels in serum (**A**) and BALF (**B**) were detected by ELISA. (**C**) TNFα, IL-6, and IL-1β mRNA expression in lungs were detected by real-time PCR. Data are presented as mean ± SEM of five mice per condition and represent one of 2 independent experiments. Statistical analyses were performed by Student’s *t* test. *, *P* < 0.05, **, *P* < 0.01.

Next, we evaluated inflammatory cytokine levels in response to *A. baumannii* infection in aged and young mice. The basal levels of TNF-α, IL-1β, and IL-6 in BALF and serum were comparable between aged and young mice. At 24 hpi, TNF-α and IL-6 levels in serum and BALF from aged mice were significantly higher than those from young mice. However, there was no significant difference of IL-1β levels in BALF and serum between infected young and aged mice (Figure 3A and B). In addition, TNF-α and IL-6 mRNA expression in the lungs from infected aged mice were significantly higher than those from infected young mice, whereas mRNA expression of IL-1β exhibited similar fold change in infected young and aged groups (Figure 3C). The data suggest aging results in a markedly local and systemic inflammation characterized by enhanced TNF-α and IL-6 expression.

### Increased inflammatory cell infiltration in BALF in aged mice after infection

**Figure 4:**
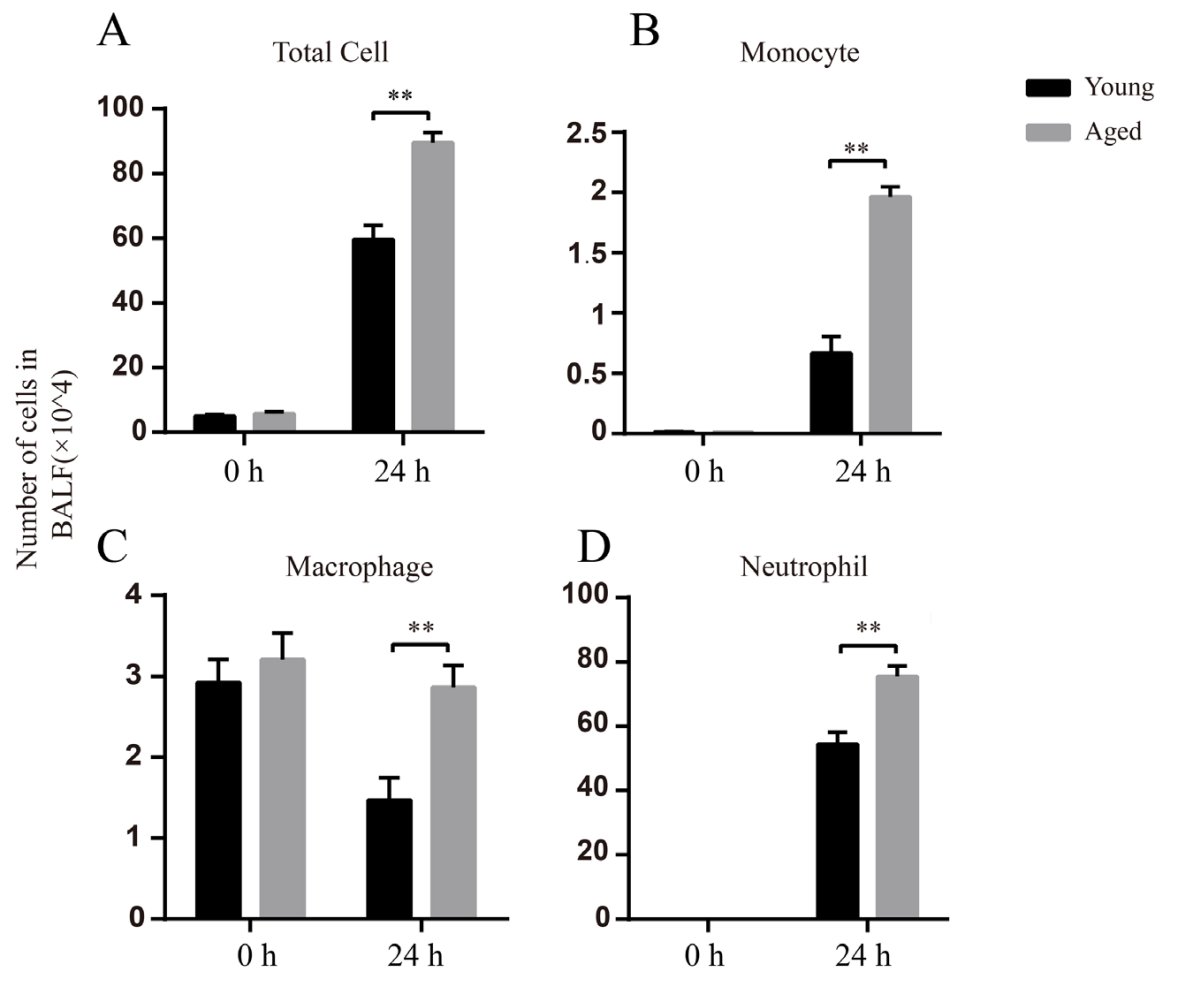
**Elevated numbers of inflammatory cells after *A.baumannii* infection in aged mice.** Young and aged mice were infected intratracheally with 5×10^6^ CFU LAC-4 and BALF were collected at 0 h and 24 hpi. Number of total cells (**A**), monocytes (**B**), macrophages (**C**), and neutrophils (**D**) in BALF of young and aged mice were determined. Data are presented as mean ± SEM of five mice per condition and represent one of 2 independent experiments. Statistical analyses were performed by Student’s *t* test. **, *P* < 0.01.

In BALF, there was no obvious difference in the total cell numbers between uninfected young and aged mice (0 h). The main cells were macrophages before infection (Figure 4C), whereas the neutrophils and monocytes were barely detected (Figure 4B and D). At 24 hpi, the total cell numbers increased in both young and aged mice compared to uninfected mice and aged mice had significantly higher total cell numbers than young mice (Figure 4A). At 24 hpi, neutrophils were the dominant cells in BALF and the numbers of neutrophils in aged mice were approximately 1.5-fold higher than those in young mice (Figure 4D). Taken together, aged mice have enhanced inflammatory cell infiltration after respiratory *A. baumannii* infection.

### Enhanced chemokine production in aged mice in response to *A. baumannii* infection

**Figure 5:**
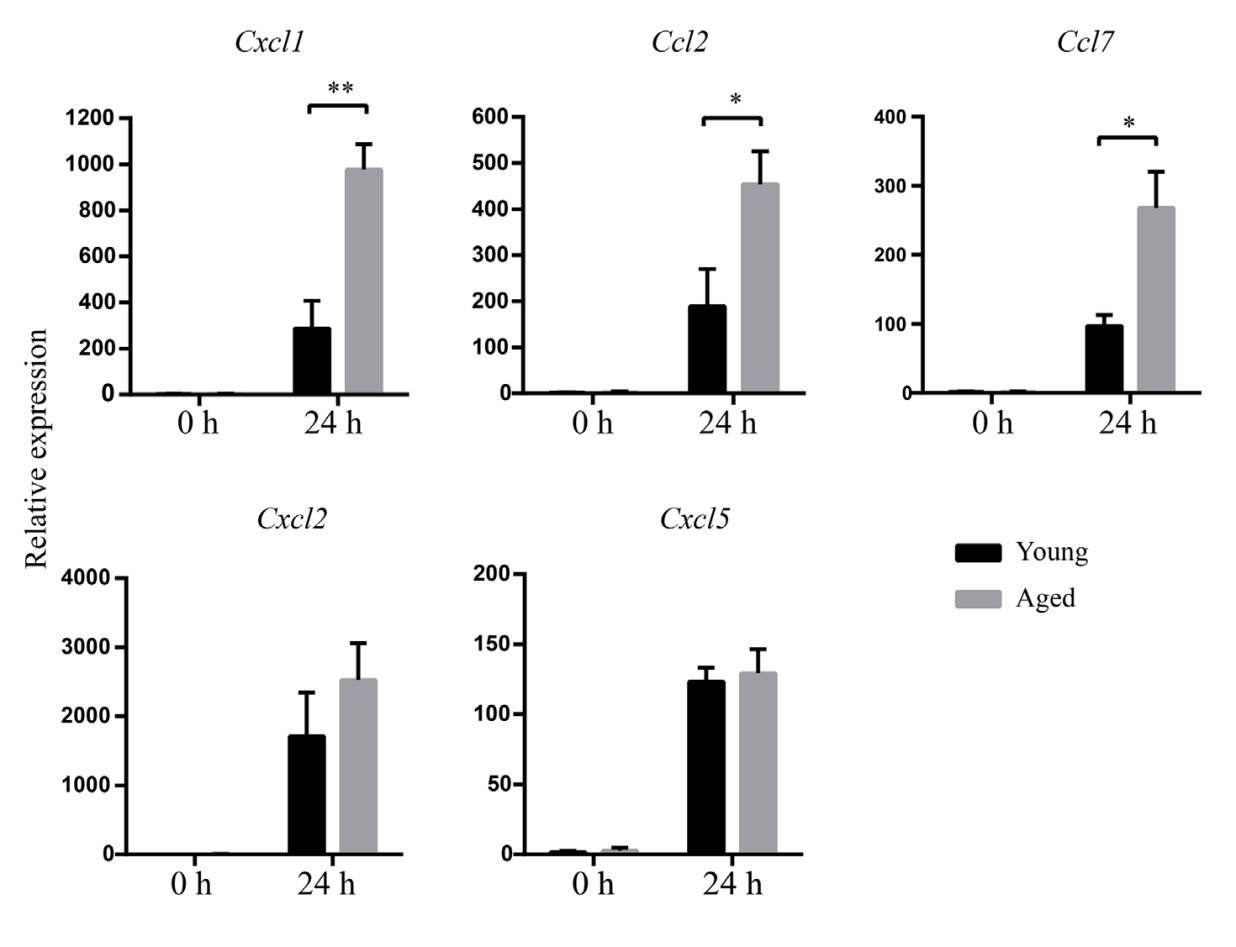
**Increased chemokines mRNA expression after *A.baumannii* infection in aged mice.** Young and aged mice were infected intratracheally with 5×10^6^ CFU of LAC-4. At 0 h and 24 hpi, mRNA in lungs was isolated. CXCL1, CCL2, CCL7, CXCL2, and CXCL5 were measured by real-time PCR. Data are presented as mean ± SEM of four or five mice per condition and represent one of 2 independent experiments. Statistical analyses were performed by Student’s *t* test. *, *P* < 0.05, **, *P* < 0.01.

To determine whether increased inflammatory cell infiltration in aged mice is associated with increased chemokine expression, mRNA levels of chemokine in lungs were detected by real-time PCR. The results showed there is no difference of chemokine mRNA expression between uninfected young and aged mice. At 24 hpi, higher mRNA expression of CXCL1, CCL2, and CCL7 in lungs were detected in aged mice than those in young mice (Figure 5). There was no obvious difference in mRNA levels of CXCL2 and CXCL5 between infected young and aged mice. The results suggest increased inflammatory cells are associated with increased levels of CXCL1, CCL2, and CCL7 expression.

### Decreased bactericidal ability of inflammatory cells in aged mice

**Figure 6:**
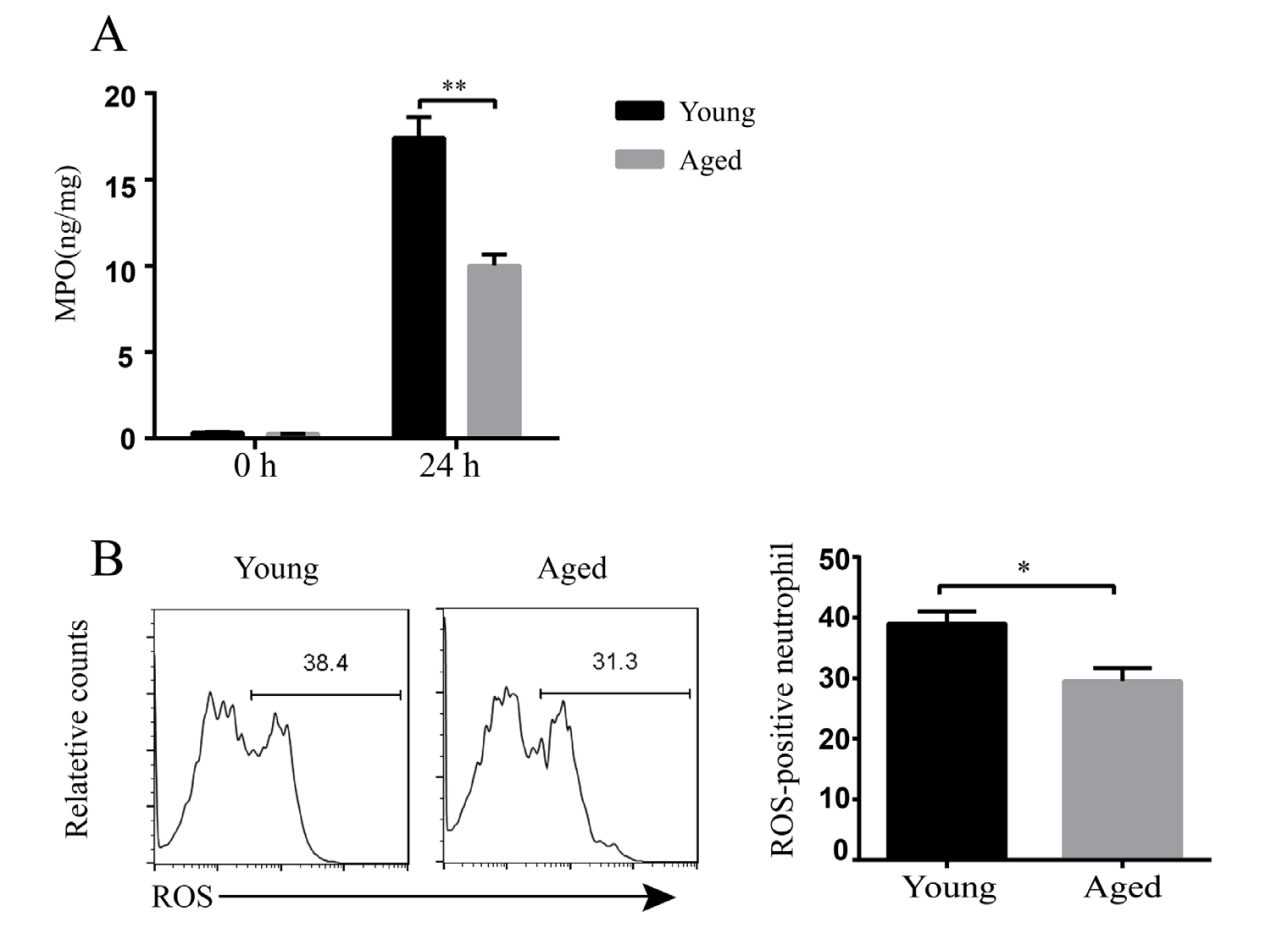
**Bactericidal ability of inflammatory cells in young and aged mice.** Young and aged mice were infected intratracheally with 5 × 10^6^ CFU of LAC-4. (**A**) MPO levels in the lung homogenate supernatants at 0 h and 24 hpi were detected by ELISA. (**B**) ROS production in neutrophils (CD11b^+^ Ly6G^+^ cells) in BALF at 24 hpi was stained by a ROS detection reagent (carboxy-H_2_DCFDA) and assessed by flow cytometry. Data are presented as mean ± SEM of four or five mice per condition and represent one of 2 independent experiments. Statistical analyses were performed by Student’s *t* test. *, *P*<0.05, **, *P*<0.01.

Usually, inflammatory cells are regarded as the first line of host defense against infection. Our data showed aged mice have stronger inflammatory response to *A. baumannii* infection. But aged mice still had higher bacterial burdens than young mice. Thus, we assumed that the bactericidal abilities of inflammatory cells in aged mice might be impaired. We detected myeloperoxidase (MPO) levels in lungs and reactive oxygen species (ROS) activity of the neutrophils. The results showed basal MPO levels in lung homogenates are similar between uninfected young and aged mice. But MPO levels at 24 hpi were significantly lower in aged mice than in young mice (Figure 6A). In addition, ROS-positive neutrophils in BLAF of aged mice were significantly lower than that in young mice after infection (Figure 6B). These results indicate the bactericidal activity of inflammatory cells is reduced with aging, which might be responsible for the enhanced susceptibility to *A. baumannii* infection.

### Decreased efficacy of antibiotic therapy against *A. baumannii* in aged mice model

**Figure 7:**
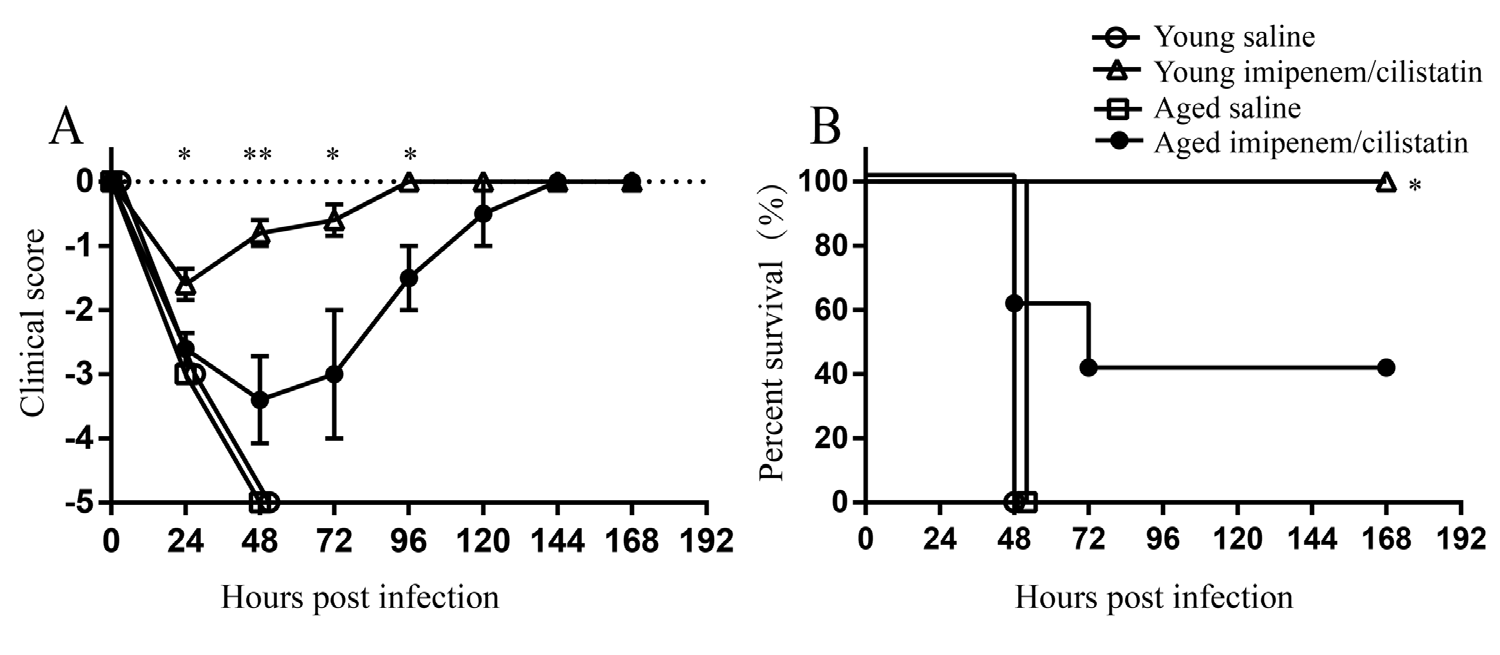
**Efficacy of imipenem/cilstatin treatment on respiratory *A.baumannii* infection.** Young and aged mice were challenged intratracheally with 1.5×10^7^ CFU of LAC-4. At 3 hpi, groups of five mice were i.p. injected with imipenem/cilstatin (20mg/20mg)/kg/day or saline twice a day. (**A**) Clinical score and (**B**) survival rate of mice were monitored for 7 days. Clinical score is expressed as means ± SEM. The data represent one of 2 independent experiments. Survival curves were compared using log-rank test. Statistical significance of clinical score was determined by Student’s *t* test. *, *P* < 0.05, **, *P* < 0.01 antibiotic-treated aged mice versus antibiotic-treated young mice.

Next, we tried to evaluate whether aging affects the efficacy of antibiotics against *A. baumannii* infection. Young and aged mice were intratracheally inoculated with lethal dose of LAC-4 (1.5×10^7^ CFU). At 3 hpi, mice were treated with imipenem/cilastatin or saline twice a day. All young and age mice treated with saline developed severe clinical signs and died 100% within 48 hours. Whereas, young mice treated with antibiotics developed mild clinical signs and 100% survived the infection. In contrast, aged mice after antibiotic treatment showed more severe clinical signs than treated young mice (Figure 7A). Only 40% of aged mice treated with antibiotics survived (Figure 7B). In conclusion, these data suggest aging reduces the efficacy of antibiotics against *A. baumannii*.

### Reduced protection of vaccination against *A. baumannii* infection in aged mice

**Figure 8:**
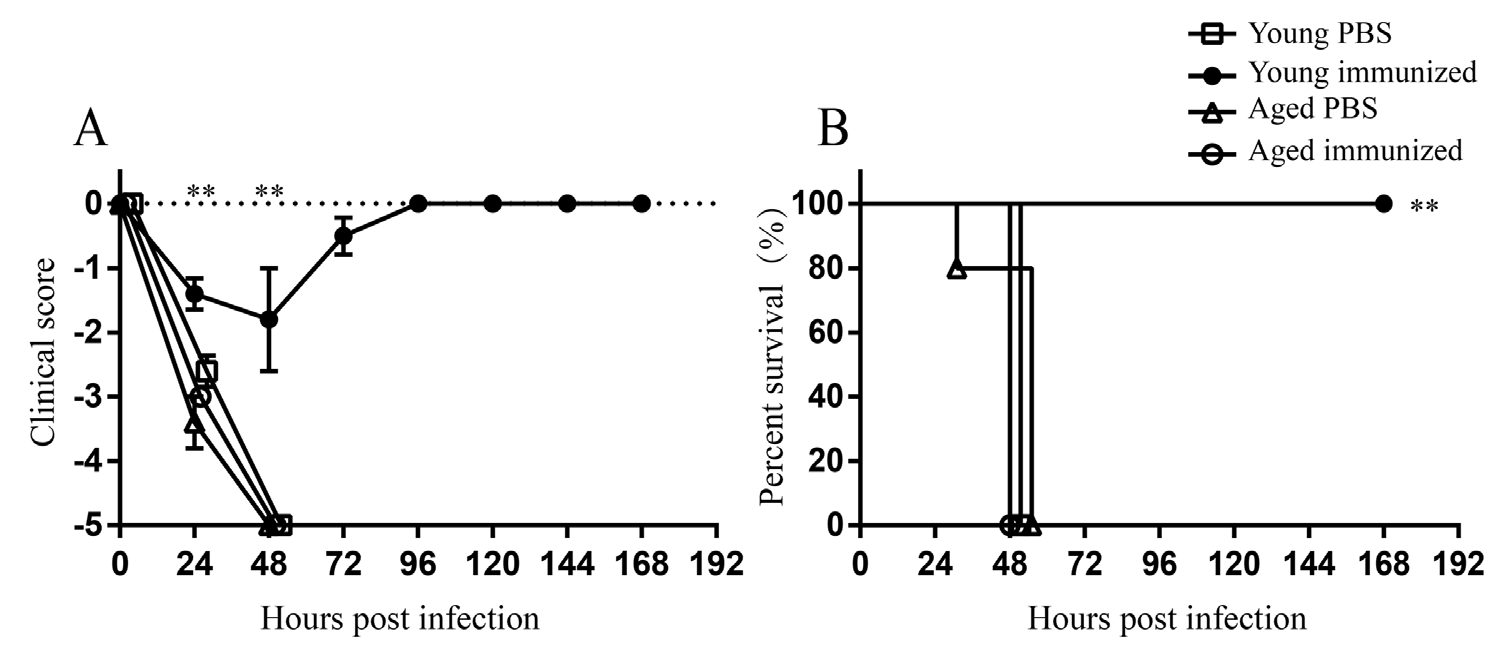
**Protection of ffLAC-4 vaccination against respiratory *A. baumannii* infection.** Groups of five young and aged mice were immunized intramuscularly with 1×10^7^ CFU of ffLAC-4 or PBS on day 0. On day 7, mice were challenged intratracheally with 1.5×10^7^ CFU of LAC-4. (**A**) Clinical score and (B) survival rate of mice were monitored for 7 days after infection. Clinical score is expressed as means ± SEM. The data represent one of 2 independent experiments. Survival curves were compared using log-rank test. Statistical significance of clinical score was determined by Student’s *t* test. **, *P* < 0.01 immunized aged mice versus immunized young mice.

Next, we evaluated whether aging affects the vaccine efficacy against *A. baumannii* infection. Both young and aged mice were immunized with formalin-fixed LAC-4 (ffLAC-4) intramuscularly and then challenged with lethal dose of LAC-4 intratracheally. Unvaccinated young and aged mice died within 48 hpi. Vaccinated young mice showed milder clinical signs compared with unvaccinated young mice (Figure 8A). The survival rate of vaccinated young mice was 100% (Figure 8B), indicating that ffLAC-4 immunization induces protective response against *A. baumannii* infection in young mice. Whereas vaccinated aged mice were very sick and moved slowly after infection, showing the similar clinical scores to unvaccinated aged mice (Figure 8A). Vaccinated aged mice all died within 48 hpi (Figure 8B). These results indicate aging significantly reduces the protection induced by vaccination.

## DISCUSSION

Aging is known to increase the risk of infections due to immune dysregulations in the elderly [13], but the effect of aging on *A. baumannii* infection is still unknown. Here, we developed a mouse model of respiratory *A. baumannii* infection in aged mice. The results show that aged mice are more susceptible to *A. baumannii* infection, since aged mice had higher mortality, increased bacterial burdens, and more severe lung injury than young mice after intratracheal infection of *A. baumannii*.

Histopathology of lung and inflammatory cell detection in BALF showed excessive inflammatory cells infiltrating in aged mice, including macrophages and neutrophils. To account for the heightened inflammatory cell infiltration, we detected the levels of some chemokines. CXCL1, CCL2, and CCL7 in the lungs were significantly increased in aged mice after infection. CXCL1 acts on receptor CXCR2 and mediates neutrophil migration to sites of inflammation [20]. CCL2 and CCL7 exhibit chemotactic activity for monocytes and regulate macrophage function through receptor CCR2 [21,22]. Therefore, the enhanced inflammatory cell infiltration is consistent with the increased levels of CXCL1, CCL2, and CCL7. Inflammatory cells including neutrophils and macrophages are first line to combat *A. baumannii* infection [23]. However, clearance of bacteria was not increased with enhanced inflammatory cell infiltration in aged mice. Therefore, we assumed that the bactericidal activity of inflammatory cells might be impaired with aging, since it has been reported that inflammatory cells including neutrophils and macrophages from aged mice have a compromised bactericidal potential [24]. MPO is a major enzyme which is present at high levels in neutrophils, monocytes, and macrophages. With H_2_O_2_, MPO generates a wide array of reactive intermediates to kill the invading bacteria, which is important for host defense [25]. The oxidative burst also plays an essential role in the rapid killing of ingested *A. baumannii* by neutrophils [26]. Our study showed MPO levels in the lung and the percentage of ROS-positive neutrophils in *A. baumannii*-infected aged mice were significantly reduced compared with those in infected young mice. These results indicate the bactericidal function of inflammatory cells is impaired with aging. This could explain why increased inflammatory cell infiltration was detected in the lung, whereas bacteria were not controlled in aged mice after infection. On the other hand, local infiltration of neutrophils and macrophages plays a critical role in the development of pathology [27,28]. The high levels of pro-inflammatory cytokines produced by these cells could exacerbate tissue damage [29]. In our model, heightened inflammatory cells infiltration along with the increased pro-inflammatory cytokines in the lungs of aged mice after *A. baumannii* infection might induce lung injury rather than clear bacteria. Taken together, the higher mortality of aged mice is probably a result of unbalance between host defense and excessive inflammatory response. However, why aging leads to the decreased bactericidal potential of inflammatory cells needs further study. So, our model provides a good model to study the underlining mechanisms of age-related changes in the immune system to *A. baumannii* infection, which could help us to design effective strategies to prevent and treatment this infection in the elderly.

It has been suggested that aging might affect the efficacies of drug and vaccine in other infections [30,31]. However, very few experiments have specifically evaluated drug or vaccine efficacies for the elderly due to the lack of appropriate aged animal models. Reproducing the clinical features after *A. baumannii* infection in aged mice provides an animal model to investigate the efficacies of drug and vaccine for the elderly. We found the effectiveness of antibiotic therapy against *A. baumannii* is obviously reduced in aged mice. The similar phenomenon is also seen in the antibiotic treatment in aged sepsis model, which showed decreased therapeutic effect of antibiotics in aged mice [32]. So, it’s unreasonable to treat the elderly with the dosage for the young and drug dosage specific for the elderly should be evaluated in suitable models.

Infections of *A. baumannii* are becoming extremely difficult to treat, since this bacterium displays multidrug-resistant or extensively drug-resistant. New antibiotics which are effective against MDR *A. baumannii* will not be available in the very near future, thus vaccination seems to be an optimal way to prevent its infection. Aged people have been suggested to be the main candidates for a future *A. baumannii* vaccine [33]. However, decreased protective effects of vaccines against seasonal influenza, varicella zoster virus, and *Streptococcus pneumonia* have been seen in the elderly [34,35]. The vaccine of ffLAC-4, a whole cell inactivated by formalin, is supposed to be a strong vaccine candidate and was 100% protective in *A. baumannii-*infected young mice (Figure 8). But vaccination with ffLAC-4 in aged mice showed no protection against *A. baumannii-*infection (Figure 8). These results suggest that the vaccine tested effective in young and adult might not exhibit same efficacy in the elderly. Currently, there is no vaccine against *A. baumannii* in the market or in clinical trials. Since aging is an important factor for *A. baumannii* infection, we should take aging into account when designing the vaccines against *A. baumannii*. Efforts to enhance immune response in the elderly would be important and clinically relevant. Several strategies have been suggested to improve the efficacy of vaccines against influenza and pneumococcal disease in the elderly, such as using stronger adjuvants, high-dose antigen, different immunization routes, or more shots [35,36]. These approaches might be good clues for *A. baumannii* vaccine development targeting the elderly. We also found that higher immunization dose and repeated immunization could protect aged mice from lethal dose of *A. baumannii* infection (data not shown). So, the suitable animal models for developing and testing novel vaccines for the elderly is crucial. The aged mice model developed here provides a suitable model to evaluate the efficacy of vaccine against *A. baumannii* in the elderly.

In conclusion, our results indicate aged mice are more susceptible to *A. baumannii* infection due to the increased inflammatory responses and impaired bactericidal function of inflammatory cells. We also applied this model to study the efficacies of antibiotics and vaccine against *A. baumannii* infection and found that the efficacies of them are both reduced in aged mice. Our study provides a novel *A. baumannii-*infected aged mice model for investigating the mechanisms by which aging affects the outcome of *A. baumannii* infection. More importantly, the aged mouse model of *A. baumannii* pneumonia is also a useful model to evaluate the efficacies of antibiotics and vaccines for the elderly.

## MATERIALS AND METHODS

### Mice

Young (6-8 weeks) and aged (18-21 months) female C57BL/6 mice were purchased from the Animal Center of Chongqing Medical University. The mice were kept under pathogen-free conditions. All procedures were approved by the Animal Ethical and Experimental Committee of the Amy Medical University.

### Pneumonia model and samples collection

A clinical *A. baumannii* strain LAC-4 was kindly provided by Professor Chen [37]. The bacteria were grown in tryptone soy broth at 37 °C. At mid-log-phase, bacteria were collected and suspended in phosphate buffer saline (PBS). Mice were anaesthetized by intraperitoneal injection of pentobarbital sodium (62.5 mg/kg of body weight) and then infected with LAC-4 in 20 μl PBS by non-invasive intratracheal inoculation under direct vision adapted as previously described [38]. The infection dose was confirmed by plating 10-fold serial dilutions on tryptone soy agar (TSA) and counting bacteria colony-forming unit (CFU). The survival rate and clinical score of mice were monitored daily for 7 days (n = 10). The clinical scores were recorded as described previously [37]: 0, normal clinical signs; -1, slightly ruffled fur but active; -2, ruffled fur, slow movement, and sick; -3, ruffled fur, hunched posture, squeezed eyes, and very sick; -4, moribund; -5, dead. At designated time, groups of five mice were sacrificed and bacterial burdens in the lung, blood, and spleen were determined. Serum was separated from blood samples and stored at -80 °C. Excised lungs were used for histopathology. Collection of BALF was performed by flushing of the lungs with 0.5 ml cold PBS for 3 times. The supernatant of BALF was obtained and stored at -80 °C. BALF cells were analyzed by flow cytometry.

### Quantitative bacteriology and histopathology

Lungs and spleens were homogenized in sterile PBS. Bacteria from organ homogenates or blood were determined by 10-fold serial dilutions and were cultured on TSA plates. CFUs were counted 18 h thereafter and results were expressed as log_10_ CFU per organ or per milliliter blood. For histopathology, excised lungs were fixed immediately in 4% formalin and embedded in paraffin. Sections were cut in 5 μm thick, stained with hematoxylin-eosin and observed by a light microscope.

### Cytokine and protein analysis

TNF-α, IL-1β, and IL-6 concentrations in serum and BALF were detected using ELISA kits (mouse TNF-α ELISA max standard set, mouse IL-1β ELISA max standard set, and mouse IL-6 ELISA max standard set, Biolegend). Serum albumin in BALF was assessed by mouse albumin ELISA quantitation set (Bethyl Laboratories). MPO levels in lung homogenates were quantified by mouse myeloperoxidase duoset ELISA Kit (R&D Systems). ELISAs were performed following the manufacturer's instructions.

### Quantitative real-time PCR

Total RNA of lungs was isolated by RNA iso Plus (Takara) and reverse transcribed with PrimeScript^™^ RT reagent Kit (Takara). Gene expression was detected using SYBR green Premix (Takara) with specific primers on CFX96 real-time PCR detection machine (Bio-Rad). The primer sequences used were as follows: TNF-α forward primer: 5′-CCTATGTCTCAGCCTCTT CTCAT-3′, and reverse primer: 5′-CACTTGGTGGTTT GCTACGA-3′; IL-1β forward primer: 5′-GGACCCC AAAAGATGAAGGGCTGC-3′, and reverse primer: 5′-GCTCTTGTTGATGTGCTGCTGCG-3′; IL-6 forward primer: 5′-CCTCTCTGCAAGAGACTTCC-3′, and reverse primer: 5′-CTCCGGACTTGTGAAGTAGG-3′; CXCL2 forward primer: 5′-AGGGCGGTCAAAAAG TTTGC-3′, and reverse primer: 5′-CAGGTACGATCC AGGCTTCC-3′; CXCL5 forward primer: 5′-TGGCA TTTCTGTTGCTGTTC-3′, and reverse primer: 5′-CACCTCCAAATTAGCGATCAA-3′; CCL2 forward primer: 5′-TTAAAAACCTGGATCGGAACCAA-3′, and reverse primer: 5′-GCATTAGCTTCAGATTTA CGGGT-3′; CCL7 forward primer: 5′-CCACCATG AGGATCTCTGC-3′, and reverse primer: 5′-TTGACA TAGCAGCATGTGGAT-3′; β-actin forward primer: 5′-GGCTGTATTCCCCTCCATCG-3′, and reverse primer: 5′-CCAGTTGGTAACAATGCCATGT-3′. The ΔΔCt method was used to calculate the relative gene expression with β-actin as the housekeep-gene. The results were expressed as relative fold changes compared to non-infected mice.

### Flow cytometric analysis

Cell suspensions from BALF were blocked with rat serum for 15 min. Cells were then stained with 50 μl cocktail of fluorophore-conjugated antibodies in the dark at 4 °C for 30 min. The monoclonal antibodies were as follows: CD45-PE/Cy7 (30-F11), CD11b-PerCP/Cy5.5 (M1/70), CD11c-APC (N418), Ly6G-FITC (1A8) (Biolegend). Labeled cells were analyzed on a BD FACSCanto™ II flow cytometer (BD Biosciences). The cell populations in BALF were identified as described previously [39]: CD45^+^CD11b^+^CD11c^−^Ly6G^+^ cells were considered to be neutrophils; CD45^+^CD11b^+^CD11c^−^Ly6G^low^cells were considered to be monocytes; CD45^+^CD11b^−^CD11c^+^Ly6G^−^ cells were considered to be macrophages.

### Intracellular ROS assay

Groups of four or five mice were infected intratracheally with *A. baumannii* and BALF cells were collected at 24 hpi. The isolated cells were resuspended in prewarmed hank's balanced salt solution (HBSS) containing 1 μM carboxy-H_2_DCFDA (Invitrogen) for 30 min at 37 °C, 5% CO_2_. For a negative control, cells were incubated with HBSS only. The reaction was stopped by moving the cells onto ice. Cells were stained for CD11b and Ly6G and analyzed by flow cytometry.

### Antibiotic treatment

Groups of five young and aged mice were intratracheally infected with 1.5×10^7^ CFU of LAC-4 and treated with imipenem /cilastatin ((20mg/20mg)/kg of body weight/day, twice a day, i.p.) or saline starting 3 h after inoculation for 3 days. Survival rate and clinical score of mice were observed for daily 7 days.

### Vaccination

The ffLAC-4 prepared as described was used as a vaccine [37]. Groups of five young and aged mice were intramuscularly immunized with 1×10^7^ CFU of ffLAC-4 in 250 μl PBS or PBS only at day 0. At day 7, the mice were intratracheally challenged with 1.5×10^7^ CFU of LAC-4 in 20 μl PBS. Survival rate and clinical score of mice were observed daily for 7 days.

### Statistical analysis

Statistical analysis was performed using the GraphPad Prism version 6.0. Survival curves were analyzed by log-rank tests. In all other studies, differences between young and aged groups were analyzed by Student’s *t* test. Values are expressed as mean ± SEM. A *p* value of less than 0.05 was considered statistically significant.

## References

[1] Wong D, Nielsen TB, Bonomo RA, Pantapalangkoor P, Luna B, Spellberg B. Clinical and pathophysiological overview of Acinetobacter infections: a century of challenges. Clin Microbiol Rev. 2017; 30:409–47. 27974412 10.1128/CMR.00058-16 PMC5217799

[2] Custovic A, Smajlovic J, Tihic N, Hadzic S, Ahmetagic S, Hadzagic H. Epidemiological monitoring of nosocomial infections caused by acinetobacter baumannii. Med Arh. 2014; 68:402–06. 10.5455/medarh.2014.68.402-406 25648217 PMC4314163

[3] Zilberberg MD, Nathanson BH, Sulham K, Fan W, Shorr AF. Multidrug resistance, inappropriate empiric therapy, and hospital mortality in Acinetobacter baumannii pneumonia and sepsis. Crit Care. 2016; 20:221. 10.1186/s13054-016-1392-4 27417949 PMC4946176

[4] Tacconelli E, Magrini N. Global priority list of antibiotic-resistant bacteria to guide research, discovery, and development of new antibiotics. http://www.who.int/medicines/publications/WHO-PPL-Short_Summary_25Feb-ET_NM_WHO.pdf?ua=1.

[5] Sunenshine RH, Wright MO, Maragakis LL, Harris AD, Song X, Hebden J, Cosgrove SE, Anderson A, Carnell J, Jernigan DB, Kleinbaum DG, Perl TM, Standiford HC, Srinivasan A. Multidrug-resistant Acinetobacter infection mortality rate and length of hospitalization. Emerg Infect Dis. 2007; 13:97–103. 10.3201/eid1301.060716 17370521 PMC2725827

[6] Maragakis LL, Perl TM. Acinetobacter baumannii: epidemiology, antimicrobial resistance, and treatment options. Clin Infect Dis. 2008; 46:1254–63. 10.1086/529198 18444865

[7] Spellberg B, Bonomo RA. The deadly impact of extreme drug resistance in Acinetobacter baumannii. Crit Care Med. 2014; 42:1289–91. 10.1097/CCM.0000000000000181 24736340 PMC4184139

[8] Huang H, Chen B, Liu G, Ran J, Lian X, Huang X, Wang N, Huang Z. A multi-center study on the risk factors of infection caused by multi-drug resistant Acinetobacter baumannii. BMC Infect Dis. 2018; 18:11. 10.1186/s12879-017-2932-5 29304746 PMC5756379

[9] Sengstock DM, Thyagarajan R, Apalara J, Mira A, Chopra T, Kaye KS. Multidrug-resistant Acinetobacter baumannii: an emerging pathogen among older adults in community hospitals and nursing homes. Clin Infect Dis. 2010; 50:1611–16. 10.1086/652759 20462357

[10] Fournier PE, Richet H, Weinstein RA. The epidemiology and control of Acinetobacter baumannii in health care facilities. Clin Infect Dis. 2006; 42:692–99. 10.1086/500202 16447117

[11] Garnacho J, Sole-Violan J, Sa-Borges M, Diaz E, Rello J. Clinical impact of pneumonia caused by Acinetobacter baumannii in intubated patients: a matched cohort study. Crit Care Med. 2003; 31:2478–82. 10.1097/01.CCM.0000089936.09573.F3 14530754

[12] Garnacho-Montero J, Ortiz-Leyba C, Fernández-Hinojosa E, Aldabó-Pallás T, Cayuela A, Marquez-Vácaro JA, Garcia-Curiel A, Jiménez-Jiménez FJ. Acinetobacter baumannii ventilator-associated pneumonia: epidemiological and clinical findings. Intensive Care Med. 2005; 31:649–55. 10.1007/s00134-005-2598-0 15785929

[13] Kline KA, Bowdish DM. Infection in an aging population. Curr Opin Microbiol. 2016; 29:63–67. 10.1016/j.mib.2015.11.003 26673958

[14] Mahbub S, Brubaker AL, Kovacs EJ. Aging of the innate immune system: an update. Curr Immunol Rev. 2011; 7:104–15. 10.2174/157339511794474181 21461315 PMC3066013

[15] Allman D, Miller JP. B cell development and receptor diversity during aging. Curr Opin Immunol. 2005; 17:463–67. 10.1016/j.coi.2005.07.002 16054808

[16] Goronzy JJ, Weyand CM. T cell development and receptor diversity during aging. Curr Opin Immunol. 2005; 17:468–75. 10.1016/j.coi.2005.07.020 16098723

[17] Toapanta FR, Ross TM. Impaired immune responses in the lungs of aged mice following influenza infection. Respir Res. 2009; 10:112. 10.1186/1465-9921-10-112 19922665 PMC2785782

[18] Weinberger B, Grubeck-Loebenstein B. Vaccines for the elderly. Clin Microbiol Infect. 2012 (Suppl 5); 18:100–08. 10.1111/j.1469-0691.2012.03944.x 22862783

[19] Shin JH, Warren CA. Collateral damage during antibiotic treatment of C. difficile infection in the aged host: insights into why recurrent disease happens. Gut Microbes. 2017; 8:504–10. 10.1080/19490976.2017.1323616 28453386 PMC5628656

[20] Sanz MJ, Kubes P. Neutrophil-active chemokines in in vivo imaging of neutrophil trafficking. Eur J Immunol. 2012; 42:278–83. 10.1002/eji.201142231 22359100

[21] Tsou CL, Peters W, Si Y, Slaymaker S, Aslanian AM, Weisberg SP, Mack M, Charo IF. Critical roles for CCR2 and MCP-3 in monocyte mobilization from bone marrow and recruitment to inflammatory sites. J Clin Invest. 2007; 117:902–09. 10.1172/JCI29919 17364026 PMC1810572

[22] Serbina NV, Shi C, Pamer EG. Monocyte-mediated immune defense against murine Listeria monocytogenes infection. Adv Immunol. 2012; 113:119–34. 10.1016/B978-0-12-394590-7.00003-8 22244581 PMC3985089

[23] García-Patiño MG, García-Contreras R, Licona-Limón P. The immune response against Acinetobacter baumannii, an emerging pathogen in nosocomial infections. Front Immunol. 2017; 8:441. 10.3389/fimmu.2017.00441 28446911 PMC5388700

[24] Kovacs EJ, Palmer JL, Fortin CF, Fülöp T Jr, Goldstein DR, Linton PJ. Aging and innate immunity in the mouse: impact of intrinsic and extrinsic factors. Trends Immunol. 2009; 30:319–24. 10.1016/j.it.2009.03.012 19541536 PMC2898122

[25] Aratani Y. Myeloperoxidase: its role for host defense, inflammation, and neutrophil function. Arch Biochem Biophys. 2018; 640:47–52. 10.1016/j.abb.2018.01.004 29336940

[26] Qiu H, Kuolee R, Harris G, Chen W. Role of NADPH phagocyte oxidase in host defense against acute respiratory Acinetobacter baumannii infection in mice. Infect Immun. 2009; 77:1015–21. 10.1128/IAI.01029-08 19103777 PMC2643620

[27] Ramaiah SK, Jaeschke H. Role of neutrophils in the pathogenesis of acute inflammatory liver injury. Toxicol Pathol. 2007; 35:757–66. 10.1080/01926230701584163 17943649

[28] Clayton KL, Garcia JV, Clements JE, Walker BD. HIV infection of macrophages: implications for pathogenesis and cure. Pathog Immun. 2017; 2:179–92. 10.20411/pai.v2i2.204 28752134 PMC5526341

[29] Chousterman BG, Swirski FK, Weber GF. Cytokine storm and sepsis disease pathogenesis. Semin Immunopathol. 2017; 39:517–28. 10.1007/s00281-017-0639-8 28555385

[30] Del Giudice G, Goronzy JJ, Grubeck-Loebenstein B, Lambert PH, Mrkvan T, Stoddard JJ, Doherty TM. Fighting against a protean enemy: immunosenescence, vaccines, and healthy aging. NPJ Aging Mech Dis. 2017; 4:1. 10.1038/s41514-017-0020-0 29285399 PMC5740164

[31] Meyers BR, Wilkinson P. Clinical pharmacokinetics of antibacterial drugs in the elderly. Implications for selection and dosage. Clin Pharmacokinet. 1989; 17:385–95. 10.2165/00003088-198917060-00003 2689039

[32] Turnbull IR, Wlzorek JJ, Osborne D, Hotchkiss RS, Coopersmith CM, Buchman TG. Effects of age on mortality and antibiotic efficacy in cecal ligation and puncture. Shock. 2003; 19:310–13. 10.1097/00024382-200304000-00003 12688540

[33] Perez F, Bonomo RA. Vaccines for Acinetobacter baumannii: thinking “out of the box”. Vaccine. 2014; 32:2537–39. 10.1016/j.vaccine.2014.03.031 24662709 PMC4028134

[34] Lefebvre JS, Lorenzo EC, Masters AR, Hopkins JW, Eaton SM, Smiley ST, Haynes L. Vaccine efficacy and T helper cell differentiation change with aging. Oncotarget. 2016; 7:33581–94. 10.18632/oncotarget.9254 27177221 PMC5085104

[35] Boraschi D, Italiani P. Immunosenescence and vaccine failure in the elderly: strategies for improving response. Immunol Lett. 2014; 162:346–53. 10.1016/j.imlet.2014.06.006 24960535

[36] Fujihashi K, Sato S, Kiyono H. Mucosal adjuvants for vaccines to control upper respiratory infections in the elderly. Exp Gerontol. 2014; 54:21–26. 10.1016/j.exger.2014.01.006 24440991 PMC3989367

[37] Harris G, Kuo Lee R, Lam CK, Kanzaki G, Patel GB, Xu HH, Chen W. A mouse model of Acinetobacter baumannii-associated pneumonia using a clinically isolated hypervirulent strain. Antimicrob Agents Chemother. 2013; 57:3601–13. 10.1128/AAC.00944-13 23689726 PMC3719758

[38] Spoelstra EN, Ince C, Koeman A, Emons VM, Brouwer LA, van Luyn MJ, Westerink BH, Remie R. A novel and simple method for endotracheal intubation of mice. Lab Anim. 2007; 41:128–35. 10.1258/002367707779399400 17234059

[39] Shepardson KM, Jhingran A, Caffrey A, Obar JJ, Suratt BT, Berwin BL, Hohl TM, Cramer RA. Myeloid derived hypoxia inducible factor 1-alpha is required for protection against pulmonary Aspergillus fumigatus infection. PLoS Pathog. 2014; 10:e1004378. 10.1371/journal.ppat.1004378 25255025 PMC4177996

